# Development of a Meander-Coil-Type Dual Magnetic Group Circumferential Magnetostrictive Guided Wave Transducer for Detecting Small Defects Hidden behind Support Structures

**DOI:** 10.3390/mi15101261

**Published:** 2024-10-15

**Authors:** Jinjie Zhou, Hang Zhang, Yuepeng Chen, Jitang Zhang

**Affiliations:** 1 School of Mechanical Engineering, North University of China, Taiyuan 030051, China; 2 Shanxi Key Laboratory of Intelligent Equipment Technology in Harsh Environment, Taiyuan 030051, China

**Keywords:** magnetostrictive patch transducer, high frequency, CSH, pipeline, circumferential, support, defect detection

## Abstract

In order to solve the problem that small defects hidden behind pipeline support parts are difficult to detect effectively in small spaces, such as offshore oil platforms, a meander-coil-type dual magnetic group circumferential magnetostrictive guided wave transducer is developed in this paper. The transducer, which consists of a coil, two sets of permanent magnets, and a magnetostrictive patch, can excite a high-frequency circumferential shear horizontal (CSH) guided wave. The energy conversion efficiency of the MPT is optimized through magnetic field simulation and experiment, and the amplitude of the defect signal is enhanced 1.9 times. The experimental results show that the MPT developed in this paper can effectively excite and receive CSH_2_ mode guided waves with a center frequency of 1.6 MHz. Compared with the traditional PPM EMAT transducer, the excitation energy of the transducer is significantly enhanced, and the defects of the 2 mm round hole at the back of the support can be effectively detected.

## 1. Introduction

Sudden oil spill accidents, such as blowout of offshore oil wells and tanker accidents, are one of the main causes of marine oil pollution. The process pipelines on offshore oil exploitation platforms are crisscrossing, and the system is complex. Due to the dangerous properties of petroleum in the process of exploitation, treatment, and transportation, the stability of equipment and pipelines is highly necessary. The internal and external components of the pipeline are under high temperatures, high pressure, and other conditions, making it easy to produce rust, perforation, corrosion, cracks, and other defects. If the defect is not detected in time, it may cause serious consequences, such as high-pressure pipe bursts, resulting in loss of life and property [1,2]. When the pipeline is erected, the support frame is set up to ensure the stability and durability of transportation. When detecting the small defects hidden behind the support, the welding seam at the support and the support structure disperse the energy of the guided wave, resulting in high detection difficulty and making it difficult to find the defects. In addition, due to the limited space of offshore oil platforms, the space of the pipeline layout in many areas is narrow, and the span of its support is relatively small. The pipeline under testing in offshore oil platforms can hardly be removed from the support for routine inspection. In order to achieve effective detection of small defects hidden behind the support, the size and accuracy of the transducer have higher requirements. Therefore, it is necessary to develop a transducer for circumferential detection of small defects hidden behind the pipeline support structure in a narrow space.

Ultrasonic guided wave nondestructive testing technology, with its advantages of long detection distance and high detection efficiency of single point excitation, has been widely used in the detection of service pipelines [3]. Lee et al. used the SH wave generated by an electromagnetic acoustic transducer (EMAT) to realize defect detection in the welding area of the pipeline and conducted an integrity evaluation of the integrity of the welding area of the pipeline [4]. Petcher et al. used SH waves to detect defects in welds, indicating that SH waves were less affected by beam steering in welds, and missed detection could be avoided as much as possible [5]. Shankar et al. staggered multiple rows of periodic permanent magnets (PPMs) in the EMAT structure, proving that the interleaving of magnets leads to the relative redistribution of Lorentz forces in the material and that the directivity of the guided wave will follow the direction of the resultant interference [6]. Shi et al. proposed a method to detect pipeline circumferential cracks by analyzing the reflection of defect edges using the SH wave generated through the PPM EMAT [7]. It can be seen that the CSH wave (horizontal shear wave) is not easily affected by gas or liquid in the propagation process, and the attenuation is small, which is very suitable for the detection of welding defects, cracks, and corrosion defects.

Therefore, some researchers used electromagnetic ultrasonic transducers to excite SH-guided waves to study the influence of the pipe support on defect detection. Andruschak et al. found that the support structure had little influence on SH wave propagation, and they used EMAT to excite the 730 kHz SH_1_ mode to successfully detect the thinning region inside of the support structure [8]. Hu et al. selected a PPM EMAT with a low-frequency CSH_0_ mode guided wave to study the hidden defects behind the support structure and successfully detected 3 mm crack defects, but its detection ability for circular hole defects below 5 mm was limited [9]. The above research shows that EMAT with SH-guided wave excitation has been applied in pipeline support inspection and can detect defects after support. However, the center of frequency of these EMATs is low, and due to the characteristics of non-contact, the ultrasonic guided wave signal generated by them is weak and the signal-to-noise ratio is low. This makes it difficult for EMAT to effectively detect small defects hidden after pipeline support, resulting in defects being missed.

Compared with the above Lorentz-force-based electromagnetic ultrasonic guided wave transducer, the magnetostrictive-force-based electromagnetic ultrasonic transducer is a kind of electromagnetic acoustic transducer that excites the ultrasonic guided wave through a high-saturation magnetostrictive material, which significantly improves the energy conversion efficiency of EMAT and expands the application range of EMAT. In addition, a magnetostrictive patch transducer (MPT) uses a high-saturation magnetostrictive strip (such as Ni, Fe-Co-V alloy, etc.) to bond with the tested part, which can generate large magnetostrictive strain and excite ultrasonic guided waves with a high signal-to-noise ratio, thus improving the detection performance of the magnetostrictive guided wave transducer [10]. In order to improve the energy conversion efficiency of the MPT, Park et al. significantly improved the energy exchange efficiency and signal-to-noise ratio of the transducer by adding a V-shaped yoke and a Z-shaped magnetostrictive patch at both ends of the magnetostrictive patch [11,12]. Zhang et al. re-study the EMAT model of SH_0_ mode generation based on magnetostriction mechanisms and improve the accuracy of MEMAT prediction of EAETE generated through SH_0_ mode guided waves [13]. Zitoun et al. studied the characteristics of guided waves generated in movable magnetostrictive sheets. Different guided wave modes could be generated by setting different dynamic and static magnetic field directions and sizes [14]. Sha et al. found that increasing the number of coil turns can improve the sensitivity of the receiving transducer and reduce the bandwidth [15]. Li et al. designed an SH-guided wave transducer based on the magnetostriction mechanism and tested different types of defects in the welds on the plate [16]. Wang et al. proposed a wideband unidirectional SH-guided wave magnetostrictive patch transducer array that can excite random unidirectional SH-guided waves in a wide frequency range [17]. Wang et al. designed and developed a multi-channel magnetostrictive transducer that can effectively excite SH mode guided waves at the bottom of the oil tank and realize guided wave imaging of SH_0_ with multiple defects in the whole domain [18].

According to the above research results, the magnetostrictive transducer has achieved a substantial improvement in detection distance and detection performance and expanded application scenarios through transducer structure optimization, excitation parameter optimization, and model optimization. However, the existing research mainly focuses on the steel plate and the axial direction of the pipeline. At present, magnetostrictive transducers have not been developed for the circumferent direction of pipelines. Therefore, for the application scenarios of pipeline defect detection in narrow spaces, such as offshore oil platforms, it is also necessary to develop a set of magnetostrictive guided wave transducers for detecting hidden defects behind supports in a circumferential direction.

In this paper, a high-frequency circumferential shear horizontal (CSH) guided wave mode magnetostrictive patch transducer is developed using a new structure of a dual magnetic group with staggered magnetic poles to detect the small defects hidden behind the pipeline support, and its energy conversion efficiency is optimized. It has the advantages of being small, having a simple structure, and having strong adaptability, and it is suitable for small spaces, such as offshore oil platforms. Compared with traditional PMM EMAT, it has higher energy conversion efficiency and detection sensitivity, and it can detect smaller defects hidden behind the support. The rest of this paper is organized as follows. The first part introduces the design and development of the transducer. The second part sets up the experimental system. In the third part, the transducer is optimized to improve the energy conversion efficiency, and the performance of the transducer is verified by detecting different sizes and types of defects in the hiding place of the pipeline support. The fourth part summarizes the work of this paper.

## 2. Designing and Developing the Transducer

### 2.1. Magnetostrictive Patch Transducer

A magnetostrictive patch transducer (MPT) is a kind of electromagnetic acoustic transducer that excites ultrasonic guided waves through a material with a high magnetostrictive coefficient. The energy conversion efficiency of EMAT is significantly improved, which can be used for the detection of non-ferromagnetic materials and non-metallic materials, and the application range of EMAT is expanded. The MPT typically includes permanent magnets, a coil, and a magnetostrictive patch. In an MPT, the magnetostrictive force is the dominant force, and the Lorentz force is ignored because the direction of the static magnetic field is parallel to the eddy current direction of the coil. The relative direction of the static magnetic field Hs and the dynamic magnetic field H_d_ of the MPT determines the generation or receiving mode of the guided wave. In the circumferential direction of the pipeline, when the static magnetic field H_s_ provided by the permanent magnet is perpendicular to the dynamic magnetic field H_d_ provided by the coil, guided waves of the CSH type are excited [19,20,21].

### 2.2. Transducer Design

The design of the magnetostrictive patch transducer for pipeline circumferential inspection requires redesigning the structure of the transducer; otherwise, the magnetic field inside of the patch will be very uneven, and the detection signal will be messy. The use of bending magnets to adapt to the surface of the pipeline makes it is necessary to select magnets of different curvatures for pipelines of different diameters, and the matching accuracy of the size of the pipeline and the transducer is sharply improved during the detection process, which increases the detection cost and makes it almost impossible to apply in engineering.

**Figure 1:**
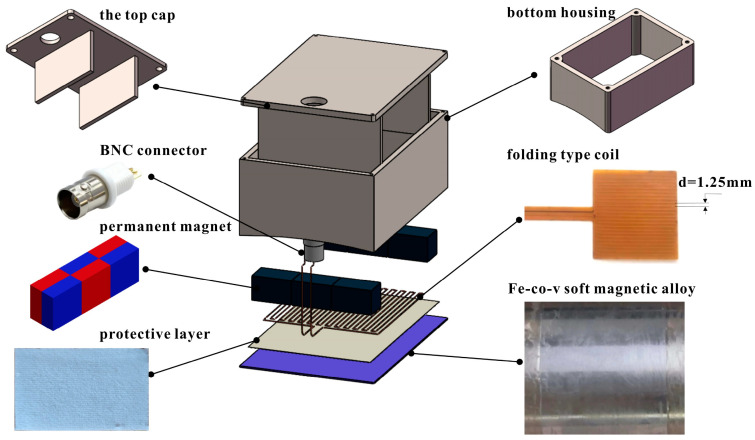
Specific structure of the transducer.

In order to solve the above problems, a meander-coil-type dual magnetic group circumferential magnetostrictive guided wave transducer is developed in this paper. The transducer, which consists of a coil, two sets of permanent magnets, and a magnetostrictive patch, can excite a high-frequency circumferential shear horizontal (CSH) guided wave. The overall transducer is a rectangular flexible multi-layer sheet structure, and the shell is finally packaged. The magnetostrictive strip is bonded to the surface of the pipeline with epoxy resin, and a meander coil is placed on the strip. Two groups of permanent magnets are placed symmetrically at the folding end of the coil, and the shell is packaged and fixed. The bottom of the housing is a flexible protective layer to prevent the coil from being damaged during the movement of the transducer, as shown in Figure 1. Due to the use of multiple magnets, the transducer can not only better fit the pipe with different pipe diameters and achieve good detection efficiency, but it also takes into account the cost and is closer to the actual engineering application. The structure of the excitation transducer and the receiving transducer is the same.

The magnetostrictive strip used in the transducer designed in this paper is an iron–cobalt–vanadium soft magnetic alloy; the geometric size (length × width × height) is 50 mm × 50 mm × 0.1 mm; the permanent magnet material is NdFeb; the residual magnetic flux density is 1.4 T; and the geometric size (length × width × height) is 10 mm × 10 mm × 7 mm. Each group of permanent magnets consists of a number of magnetic blocks arranged successively along the circumference of the pipeline, and the poles of the adjacent two magnetic blocks are alternately arranged. The meander coil is a double-layer PCB coil, the spacing d is 1.25 mm, the coil wire is 0.25 mm by 0.1 mm, and the number of turns is 24. The meander coil adopts a double-layer structure, and the upper coil current and the lower coil current direction are the same. This design can improve the amplitude of the dynamic magnetic field of the coil. Attention should be paid to keeping the folding direction of the meander coil consistent with the circumference of the pipeline.

Parts of the performance parameters of the materials used in the transducers and the pipelines in the following paper are shown in Table 1.

**Figure 2:**
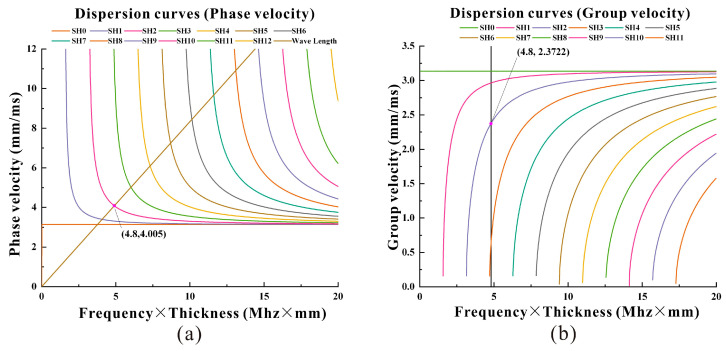
The theoretical dispersion curve of the SH wave: (**a**) phase velocity dispersion curve; (**b**) group velocity dispersion curve.

The dispersion characteristics of the guided wave should be considered when designing a guided wave transducer. The theoretical dispersion curve of the CSH wave on the steel pipe is similar to the theoretical dispersion curve of the SH wave guide steel plate. The theoretical dispersion curve of the SH waveguide in a carbon steel plate is shown in Figure 2.

According to the principle of the superposition of waves [22,23], the center distance d between adjacent wires of this coil is 1.25 mm, which is half the wavelength, so the SH guide wavelength λ is 2.5 mm. The thickness of the measured steel pipe is 3 mm. According to the calculated dispersion curve, the transducer selects a five-period Hamming window modulation signal with a center frequency of 1.6 MHz as the excitation signal, as shown in Figure 2. Through the dispersion curve, by choosing the appropriate center frequency and window width, the transducer can excite the guided wave of the CSH_2_ mode [24]. Because the guided wave excited by the transducer designed in this paper belongs to a high-frequency guided wave, the high-frequency signal in the magnetostrictive patch transducer has a wake phenomenon [25]. Therefore, the thickness of the magnetostrictive patch of the transducer used in this paper is excessively smoothed through grinding and thinning in the circumferent edge area, which effectively reduces the wake phenomenon of high-frequency signals [26].

**Figure 3:**
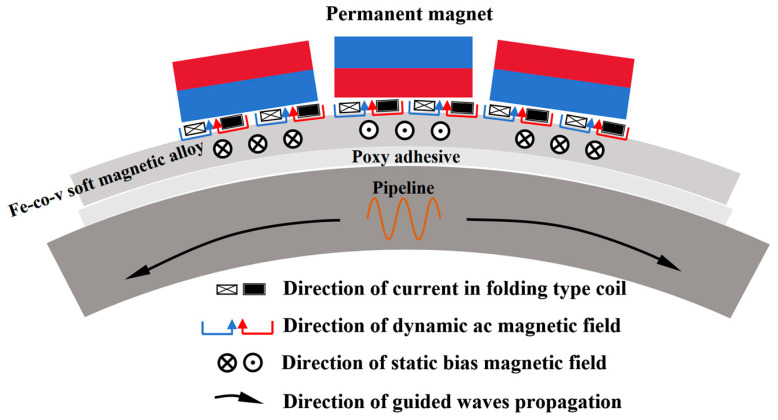
Transducer working principle diagram.

The specific working principle of the magnetostrictive high-frequency guided wave transducer designed in this paper is shown in Figure 3. A five-period Hamming window modulation signal is applied to the meander coil. Due to the skin effect, the high-frequency dynamic magnetic field H_d_ formed on the circumferential direction of the surface of the magnetostrictive patch is vertically superimposed with the static biased magnetic field Hs provided by the permanent magnet. Thus, a periodic fluctuation of magnetostrictive force is generated, and the deformation is transferred to the measured pipe in the form of mechanical coupling, forming a high-frequency electromagnetic ultrasonic guided wave based on the horizontal shear mode of circumferential propagation of the magnetostrictive effect. The guided wave will be reflected when it encounters a defect in the pipe. When the echo signal passes through the magnetostrictive guided wave receiving transducer, based on the inverse magnetostrictive effect, the elastic strain of the patch will be converted into the change of its internal magnetic field, and an electrical signal will be generated in the receiving coil. After the electrical signal is collected and saved by the collector, the waveform data are processed, and the defect location is analyzed. 

## 3. Experimental System Construction

**Figure 4:**
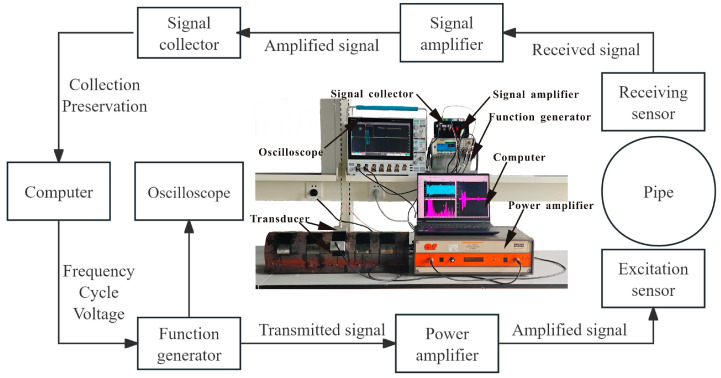
Electromagnetic ultrasonic high-order SH wave mode experimental system and schematic diagram of experimental operation.

The electromagnetic ultrasonic high-frequency CSH wave mode experimental system built in this paper mainly includes two parts: excitation and reception. The excitation part is composed of a function generator, a power amplifier, and an excitation transducer. The receiving part is composed of a receiving transducer, a signal amplifier, and a signal collector. One of the magnetostrictive guided wave transducers is used as the excitation transducer, and the other is used as the receiving transducer. The excitation transducer and the receiving transducer are arranged in a horizontal shape along the circumferential direction of the pipeline and connected to the excitation port of the power amplifier and the receiving port of the signal amplifier through the BNC connector. The running flow of the test system and the physical drawings are shown in Figure 4. The computer generates an exciting signal and inputs it into the function generator. After it is transmitted by the function generator and amplified by the power amplifier, the signal is transmitted to the excitation transducer, and the ultrasonic wave is excited in the specimen. The ultrasonic guided wave is reflected when encountering pipe defects. The magnetostrictive guided wave-receiving transducer receives ultrasonic signals in the specimen based on the inverse magnetostrictive effect. After being amplified by the signal amplifier and collected by the signal collector, the transducer is fed back to the computer to receive and store the signals and convert the digital signals into time domain signal graphs, through which the defect information of circumferential detection can be judged.

## 4. Discussion

### 4.1. Optimization of the Number of Rows of Magnets in the Magnetic Group

When magnetostrictive materials work in alternating magnetic fields, the frequency doubling phenomenon will seriously affect the sensitivity and measurement accuracy of the transducer. In this study, permanent magnets were used to add a suitable biased magnetic field to the magnetic circuit so that the output frequency of the transducer and the input frequency were consistent. In general, magnetostriction has a nonlinear relationship with magnetic fields. By setting the biased magnetic field and the appropriate dynamic magnetic field, not only can the frequency doubling phenomenon be eliminated, but it can also keep the transducer in the favorable linear working area to achieve the maximum linear oscillation.

**Figure 5:**
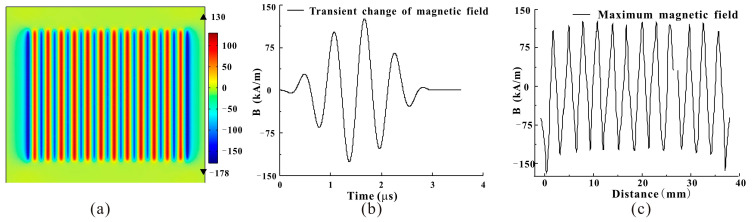
Magnetic field simulation in magnetostrictive patches. (**a**) Schematic diagram of dynamic magnetic field changes. (**b**) Dynamic magnetic field transient change curve. (**c**) Maximum magnetic field curve.

Therefore, it is necessary to determine the excitation voltage to determine the range of the dynamic magnetic field. When the excitation voltage is at the rated voltage of the coil of 250 V, it can be seen from the simulation that the dynamic magnetic field generated in the magnetostrictive patch has a small variation range and will not exceed the linear working area, as shown in Figure 5. Then, it is only necessary to select a suitable static biased magnetic field so that the magnetostrictive strain is in the linear working region; that is, the appropriate magnetostrictive strain value can be obtained. 

**Figure 6:**
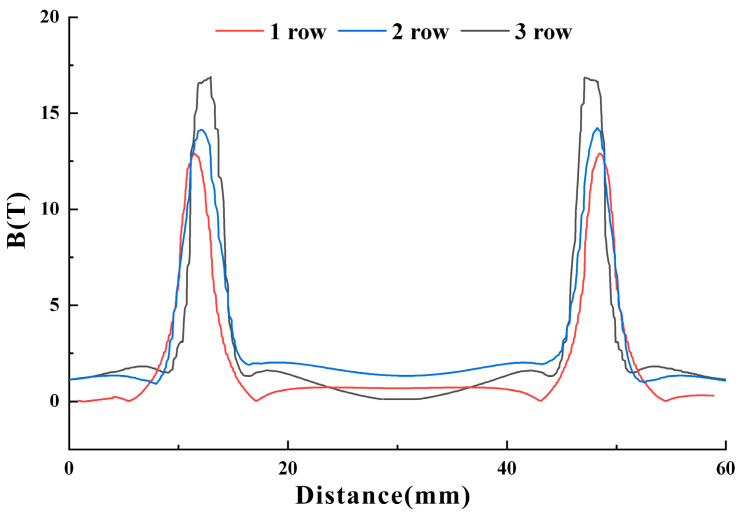
Simulation results of the influence of different rows of magnets on a static biased magnetic field in a magnetostrictive patch.

According to the Weidmann effect, the application of a biased magnetic field can enhance the output performance of a transducer, simultaneously facilitating operation within a more optimal linear working area. Next, the magnetic field intensity of the biased magnetic field will be preliminarily studied through simulation. The magnetostrictive patch area that generates the guided wave is located in the middle of the two groups of magnets, and the width and height of the two groups of magnets are 10 mm × 10 mm. By increasing the number of magnets in each group, the influence of the number of magnets on the field strength and uniformity of the biased static field in the magnetostrictive patch was studied. The permanent magnet length l is maintained at a fixed value of 3 mm, and the number is increased from one per group to three per group. The simulation results show that when the number of magnets is three, the magnetic field intensity distribution in the magnetostrictive patch is obviously not uniform, as shown in Figure 6. In the case of a single or double row of magnets, the magnetic field distribution is observed to be relatively uniform. The simulation results show that the energy conversion efficiency of the transducer can be adjusted by adjusting the row number of permanent magnets to change the intensity and uniformity of the biased magnetic field in the magnetostrictive patch. Next, we will verify experimentally that the energy conversion efficiency of the MPT can be optimized by adjusting the number of rows.

**Figure 7:**
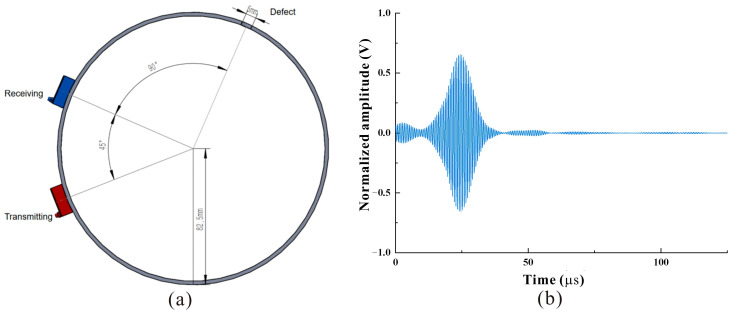
(**a**)Transducer and pipeline defect location. (**b**) Direct wave signal.

In the experiment, the permanent magnet length l remained fixed at 3 mm, and the number of permanent magnets increased from one in each group to three in each group. We recorded the amplitude of the defect signal corresponding to different rows. The 5 mm circular hole defect distribution and the transducer arrangement on the pipeline are shown in Figure 7. The time to direct signal is about 25 μs, and the corresponding sound speed is 2322 m/s. These results are basically consistent with the theoretical values of CSH_2_ mode sound velocity. The experimental results show that the transducer can excite and receive CSH_2_ mode guided waves in the pipeline.

**Figure 8:**
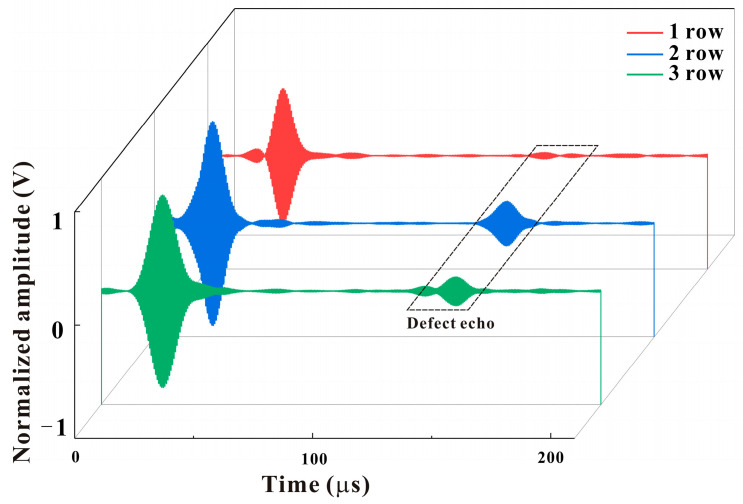
Comparison of defect detection signals of transducers with different rows of magnets.

The experimental results of defect signals detected by transducers with different rows of magnets are compared, as shown in Figure 8. When the transducer is placed in the specified experimental position, the two wave packets shown in Figure 8 are a direct signal and a defect echo signal. By examining the correlation between the defect signal and the number of rows of magnets, it can be observed that when a single row of magnets is employed, the amplitude of the defect signal is relatively modest at only 0.028 V. In contrast, when two or three rows of magnets were used, the amplitude of the signal was larger, with magnitudes of 0.188 V and 0.116 V, respectively. However, the detection signal of the transducer utilizing three rows of magnets exhibited partial clutter. This phenomenon can be attributed to the fact that when the number of rows of magnets is three, the magnetic field intensity distribution in the simulated magnetostrictive patch is demonstrably non-uniform. Accordingly, the transducer comprising two rows of magnets is selected for further investigation in this study.

### 4.2. Lift and Magnet Size Optimization

**Figure 9:**
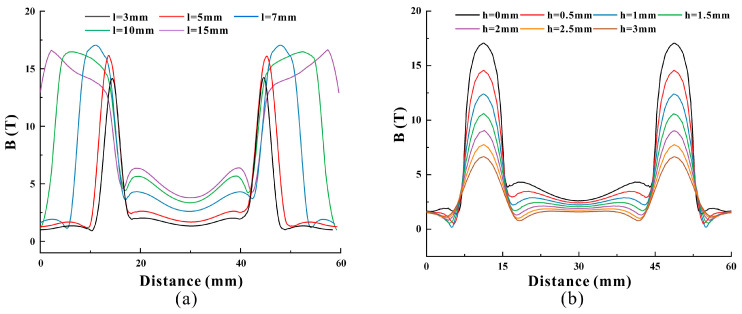
Simulation results of the static biased magnetic field of the magnetostrictive patch. (**a**) The effect of adjusting the magnet size on the static magnetic field. (**b**) The effect of adjusting the lifting distance on the static magnetic field.

In order to study the effect of the geometric parameters of the permanent magnet on the strength of the biased static magnetic field in the magnetostrictive patch, the length l of the permanent magnet was increased from 3 mm to 15 mm. As the magnet length decreases, the magnetic induction intensity of the biased static field in the magnetostrictive patch where the excitation coil is located gradually decreases, and the magnetic field’s distribution becomes more uniform. The simulation results are shown in Figure 9a. 

In order to study the effect of the lifting distance of the permanent magnet on the strength of the biased static magnetic field in the magnetostrictive patch, the lifting distance h of the permanent magnet was initially 0 mm, and then it gradually increased from 0 mm to 3 mm with a step length of 0.5 mm. When the lifting distance changes, the permanent magnet length l remains fixed at 7 mm. With the increase in distance from the permanent magnet, the biased magnetic field’s intensity in the magnetostrictive patch where the coil is located decreases, and the magnetic field’s distribution becomes more uniform, as shown in Figure 9b. The biased magnetic field’s strength and the uniformity of the magnetic field can also be adjusted by changing the distance of the permanent magnet. Next, we will experimentally verify that adjusting the magnet size and the lifting distance can change the biased field’s strength and field uniformity, thereby optimizing the energy conversion efficiency of the MPT.

**Figure 10:**
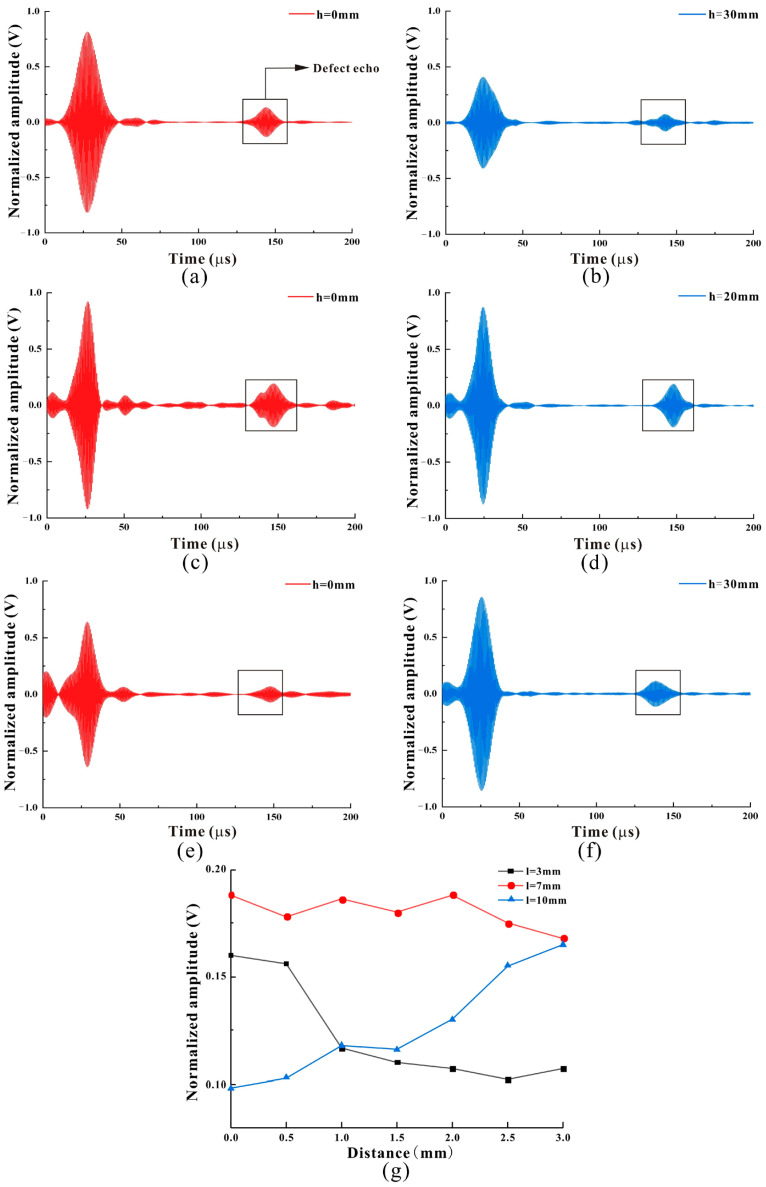
The received signal: (**a**,**c**,**e**) are signals received by the transducer when the 3 mm, 7 mm, and 10 mm magnets are not lifted. (**b**) The signal received by the transducer at the maximum lift of the 3 mm magnet; (**d**,**f**) are the best signals received by transducers with 7 mm and 10 mm magnets. (**g**) Defect detection signals received by transducers with different magnet sizes and lift distances.

The defect distribution and the transducer layout of the pipeline are the same as in 4.1. The number of rows of magnets is set to two by lifting the magnets, and the magnets with the permanent magnet size l of 3 mm, 7 mm, and 10 mm are lifted. The starting distance of the permanent magnet is 0 mm at the beginning, and then the step size of 0.5 mm is gradually increased from 0 mm to 3 mm. The corresponding received signal images of different lifts are recorded, and the results are shown in Figure 10g. As the size l of the permanent magnet is 3 mm, the signal received by the coil gradually decreases as the lifting distance of the permanent magnet increases. The received signal has a maximum amplitude of 0.16 V and a minimum amplitude of 0.102 V, as shown in Figure 10a,b. Moreover, the waveform received by the coil also changes when the lifting distance is 30 mm, which is because the biased magnetic field gradually decreases with the lifting distance of the permanent magnet. The working interval of the MPT enters the nonlinear region.

As the size of the permanent magnet l is 7 mm and the lifting distance of the permanent magnet increases, the signal received by the coil changes slightly, which is due to the large biased magnetic field provided by the 7 mm permanent magnet. The received signal has a maximum amplitude of 0.188 V and a minimum amplitude of 0.165 V, as shown in Figure 10c,d. In the process of lifting a permanent magnet, although the biased magnetic field decreases, the transducer still works in the linear region, so the overall amplitude changes little. The waveform received by the transducer is also relatively stable.

As the size of the permanent magnet l is 10 mm, the signal received by the coil gradually increases with the increase in the lifting distance of the permanent magnet. The received signal has a maximum amplitude of 0.165 V and a minimum amplitude of 0.098 V, as shown in Figure 10e,f. This is because the biased magnetic field provided by the 10 mm permanent magnet is large, which makes the transducer work in the nonlinear range when it is not lifted, so the waveform of the received signal is small and unstable. In the process of lifting the permanent magnet, with the decrease of the biased magnetic field, the working area of the transducer returns to the linear interval, and the waveform and the amplitude of the received signal are relatively stable.

Through the overall comparison of Figure 10g, it can be observed that when the permanent magnet size l is 7 mm and 10 mm, both without lifting, the overall waveform is more chaotic than that after lifting, mainly because the biased magnetic field provided by the magnet without lifting is not uniform. In this part of the experiment, we change the distance and size of the permanent magnet, adjust the strength of the biased magnetic field and the uniformity of the magnetic field, and optimize the signal-to-noise ratio and the amplitude of the detected signal. The maximum amplitude of the signal is 1.9 times that of the minimum amplitude.

### 4.3. Strip Optimization Selection

**Figure 11:**
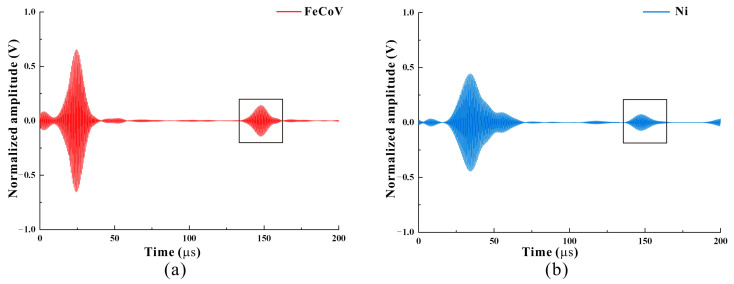
The received signal. (**a**) The signal received by the transducer when the magnetostrictive material is FeCoV. (**b**) The signal received by the transducer when the magnetostrictive material is Ni.

In this paper, a comparative study of iron, cobalt, and nickel belts is carried out. Under the above optimization results, as shown in Figure 11, it can be seen from the normalized signal that the MPT defect wave signal using a Fe-Co-V strip is twice that using a nickel band MPT defect wave signal, and its signal amplitude is 0.188 V. This phenomenon is mainly determined by the magnetostrictive strain variable $\lambda_{s}$ of the two materials with a $\lambda_{s}$ of Ni ≈ 40 ppm. The $\lambda_{s}$ of Fe-Co-V ≈ 100 ppm; in the case that both are in the best linear working area, the signal generated by Fe-Co-V material is greater than Ni. Therefore, the magnetostrictive material of the MPT selection designed in this paper is an iron–cobalt–vanadium soft magnetic alloy.

### 4.4. Experimental Verification of Transducer Performance

**Figure 12:**
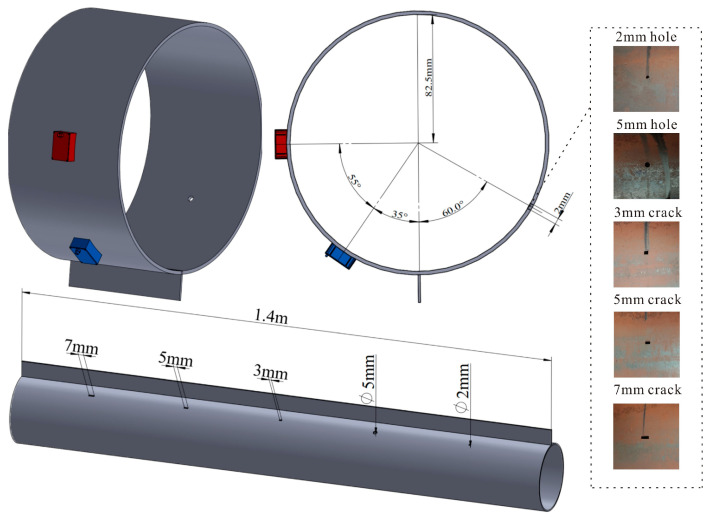
Schematic diagram of transducer layout and pipeline defect location.

The support structure of the experimental pipeline is welded by a steel plate of the same material with a length of 1.4 m, a width of 30 mm, and a thickness of 3 mm. After the support structure was completed, defects of different types and sizes were designed, with cracks of 3 mm, 5 mm, and 7 mm in length and the same width of 3 mm. The diameter Φ of circular hole defects is 5 mm and 2 mm, all defects are through holes, and the axial distance of each defect is 200 mm. The defect distribution and transducer layout on the pipeline are shown in Figure 12.

**Figure 13:**
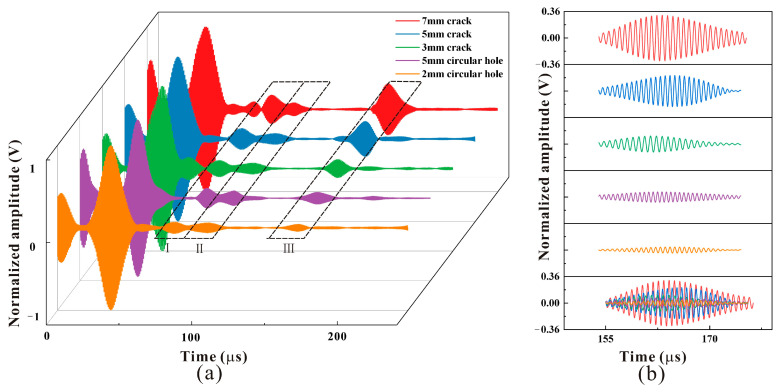
The received signal. (**a**) Detection signals for defects of different sizes and types. (**b**) Defect echo signals.

In the experiment, the MPT received the normalized signals of three crack defects and two round hole defects and drew the amplified defect signals, respectively, as shown in Figure 13. The guided waves will reflect off of the welds of the supporting structure, and they will also reflect back when they come into contact with the bottom end face. Therefore, there are clearly three wave packets in the signal diagram, namely, the weld echo at the support (wave packet I), the echo at the bottom of the support (wave packet II), and the defect echo (wave packet III). According to the relative position of the defect and the transducer in Figure 12, it is calculated that the time to receive the defect echo should be about 165 μs. By analyzing and enlarging Figure 13, the area where the defect is located during the experiment is 160 μs~170 μs. It is basically consistent with the calculated results.

In this experiment, the detection of cracks of different sizes hidden behind the support of the pipeline was studied. When the guided wave passed through the support structure, part of the energy was reflected by the weld at the support, and another part of the energy was propagated inside of the support structure after passing through the weld. As a result, the guided wave energy passing through the stent is severely weakened, resulting in some transducers being unable to accurately locate and quantify defects during detection. As seen in Figure 13b, among them, the return amplitude of the 7 mm crack defect is 0.307 V, that of the 5 mm defect is 0.210 V, and that of the 3 mm defect is 0.104 V. Compared with the 7 mm crack, the amplitude of the 3 mm crack defect is reduced by about 2/3, but the echo of the 3 mm defect is still clearly visible, and the signal-to-noise ratio is good. When the size of the defect increases, the amplitude of the detection signal for the defect after the support increases. When the size of the defect decreases, the amplitude of the signal also decreases, which indicates that the method can effectively detect the defect after the support.

Round hole defects with 5 mm diameter and 2 mm diameter were set and compared with 5 mm crack defects. Defect signals extracted for comparison are shown in Figure 13b. The return amplitude of the 5 mm crack defect was 0.210 V, and that of the 5 mm round hole defect was 0.070 V. The amplitude of the 5 mm circular hole defects is reduced by about 2/3 times compared to 5 mm crack defects. The analysis of the reasons shows that when the plane defect of the guided wave is encountered, the reflection and diffraction directions of the wave are concentrated, and the echo energy is concentrated, so the received reflected echo energy is strong. In the case of camber defects, CSH waves will reflect and diffract when interacting with the defects, resulting in a more chaotic propagation direction and more dispersed echo energy than the plane, and a lower effective feedback amplitude is received. Comparing the defects of 5 mm round holes and 2 mm round holes, it can be seen that the amplitude of the back wave of 2 mm round holes is 0.040 V, which is reduced by about 1/2.

The EMAT is able to excite and receive elastic waves in solids by utilizing Lorentz forces and magnetostriction mechanisms [27,28]. A traditional PPM EMAT based on Lorentz forces generates elastic waves by inducing eddy currents in a solid. Due to its non-contact characteristics, it has the disadvantages of a weak guided wave signal and a low signal-to-noise ratio [29,30,31]. A magnetostriction-based EMAT excites elastic waves through the piezomagnetic effect and can generate guided wave signals with a high signal-to-noise ratio [30,32]. This part will compare the detection signals of the MPT EMAT designed in this paper with those of the those of the PPM EMAT.

**Figure 14:**
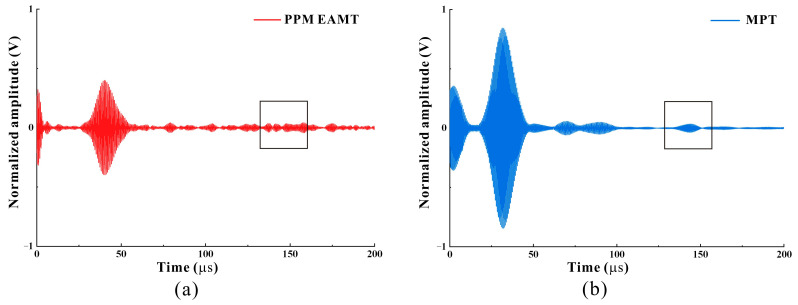
The received signal. (**a**) Received signal from the PPM EMAT. (**b**) Received signal of the MPT.

The results of PPM EMAT detection and MPT EMAT detection are normalized, as shown in Figure 14. Compared with the peak signal at the defect, it can be seen that the amplitude of the detection signal of the MPT is about twice that of the PPM EMAT, and the detection accuracy and signal-to-noise ratio of the PPM EMAT are weaker than those of the MPT designed in this paper. When detecting 2 mm round hole defects, the waveform at the theoretical position of the 2 mm hole defect in the black box in Figure 14a is very messy. The detection result of the PPM EMAT is almost unrecognizable, while the detection result of the MPT is still clearly visible. As mentioned above, the amplitude of the MPT when detecting a 2 mm round hole is still 0.040 V. The above comparison results prove that the transducer designed in this paper has good performance in circum-directional detection. For different sizes and types of defects hidden behind the support structure, the detection accuracy and signal-to-noise ratio are higher than those of PPM EMAT transducers of the same volume.

## 5. Conclusions

The pipeline structure of offshore oil production platform is complicated, which puts forward higher requirements for pipeline inspection in narrow spaces. In view of the difficulty of effectively using the existing means to detect small defects after support in the circumferential direction, this paper proposes a meander-coil-type dual magnetic group circumferential magnetostrictive guided wave transducer based on the principle of magnetostriction. The transducer structure is optimized through simulation and experimentation, and the reliability of the transducer detection is verified through comparative experiments. The following conclusions can be drawn:

(1) Based on the transducer designed on the principle of magnetostriction, a simulation system for a biased magnetic field and a dynamic magnetic field is established. The results show that the biased magnetic field in the magnetostrictive strip can be changed by adjusting the row number, the size, and the lift distance of permanent magnets in the magnetic group so as to adjust the strain variable of the strip, achieve the adjustment of the energy conversion efficiency of the transducer, and determine the optimal biased magnetic field. By adjusting the alternating current in the coil, the dynamic magnetic field in the magnetostrictive strip is changed, and the optimal range of the dynamic magnetic field is determined. Therefore, the transducer is in the best state of energy conversion efficiency, and the validity of this conclusion is verified through experiments.
(2) Through the establishment of a support structure hidden defect detection experiment system, the results show that the developed transducer can effectively excite CSH_2_ mode guided waves and clearly and reliably detect different sizes and types of defects. The reliability of the transducer for quantitative analysis of small defects hidden behind the supporting structure is proved in circumferential detection.
(3) Through the comparison experiment between the magnetostrictive transducer and the EMAT transducer, the results show that the MPT transducer designed in this paper can detect the tiny defects hidden behind the support structure, while the EMAT transducer finds it difficult to detect such small defects effectively, which proves that the magnetostrictive transducer designed in this paper has higher energy conversion efficiency and detection sensitivity.

## Figures and Tables

**Table 1:** Part of the performance parameters is related to the transducer’s patch material Fe-Co-V, nickel, and the carbon steel material used in the pipeline.

Material	Fe-Co-V	Ni	Q235
Density (kg/m^3^)	8120	8880	7850
Modulus of elasticity (GPa)	216	207	210
Poisson’s ratio	0.27~0.31	0.31	0.304
Electrical resistivity (μΩ·m)	0.27	0.684	0.14
Saturation magnetic induction (T)	2.38	0.7	
Saturation magnetostrictive coefficient (ppm)	100 ppm	40 ppm	

## Data Availability

Data are contained within the article.
